# Supplementary material for: Cytosolic thioredoxin reductase 1 is required for correct disulfide formation in the ER
Source: EMBO J. 2017 Jan 16;36(5):693–702. doi: 10.15252/embj.201695336 (PMC5331760; doi:10.15252/embj.201695336)
Supplement: Supplementary file 1 — Expanded View Figures PDF [file EMBJ-36-693-s001.pdf]

Expanded View Figures

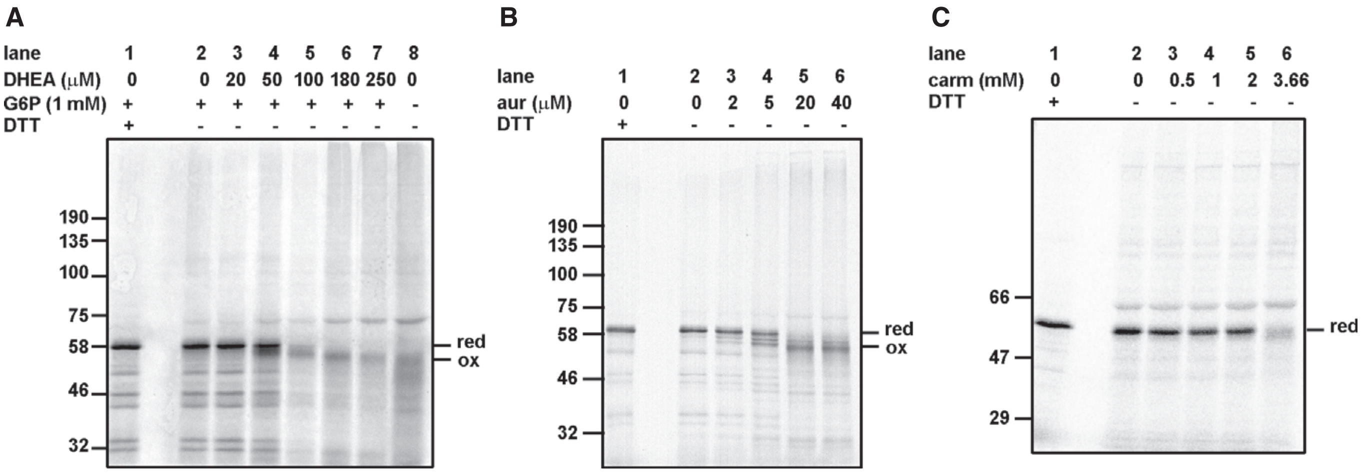

Figure EV1. Inhibition of G6PDH or TrxR1 but not GR allows disulfide formation in influenza virus HA in the presence of G6P.

A–C Cell-free translation of influenza virus HA was carried out in the presence of G6P and increasing concentrations of (A) DHEA, (B) auranofin (aur), or (C) carmustine (carm) as indicated. Translation products were separated after prior reduction with DTT (lane 1) or without reduction (A, lanes 2–8 or B and C, lanes 2–6).

Source data are available online for this figure.

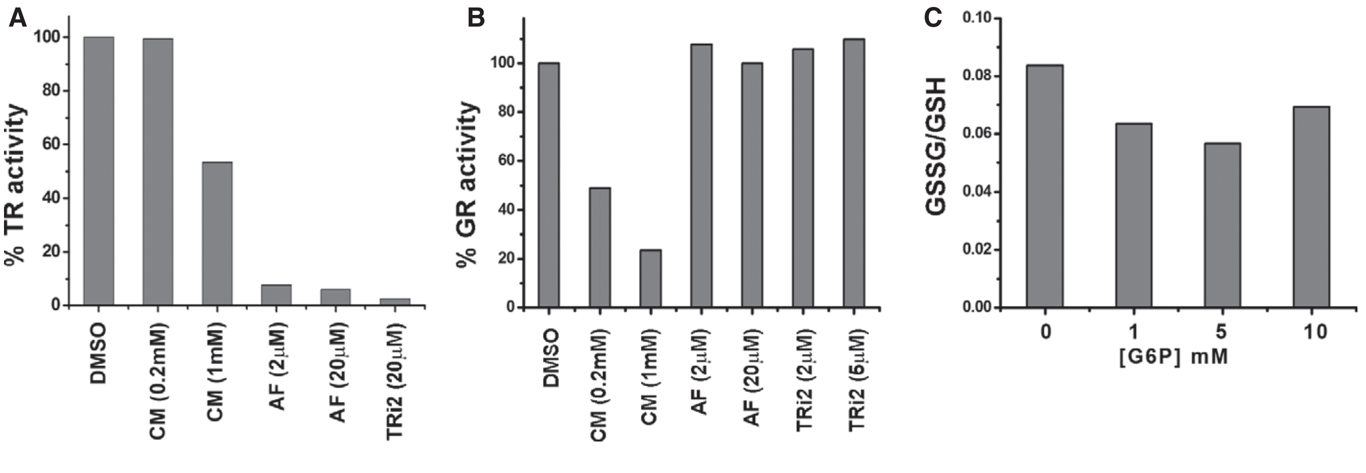

Figure EV2. Inhibition of purified TrxR1 or GR with auranofin or carmustine and ratio of GSSG/GSH before and after G6P addition.

A, B The % inhibition of purified human TrxR1 or GR was measured following addition of either carmustine, auranofin, or TRi2 at the indicated concentrations. The experiment was carried out in triplicate with similar results.

C The ratio of GSSG/GSH was calculated in a reticulocyte lysate either before or after addition of G6P at the indicated concentration. The ratios are calculated from the average values for [GSH] or [GSSG] from three determinations.

Source data are available online for this figure.

© 2017 The Authors
