# Supplementary figures and images for: Cytosolic thioredoxin reductase 1 is required for correct disulfide formation in the ER
Source: EMBO J. 2017 Jan 16;36(5):693–702. doi: 10.15252/embj.201695336 (PMC5331760; doi:10.15252/embj.201695336)

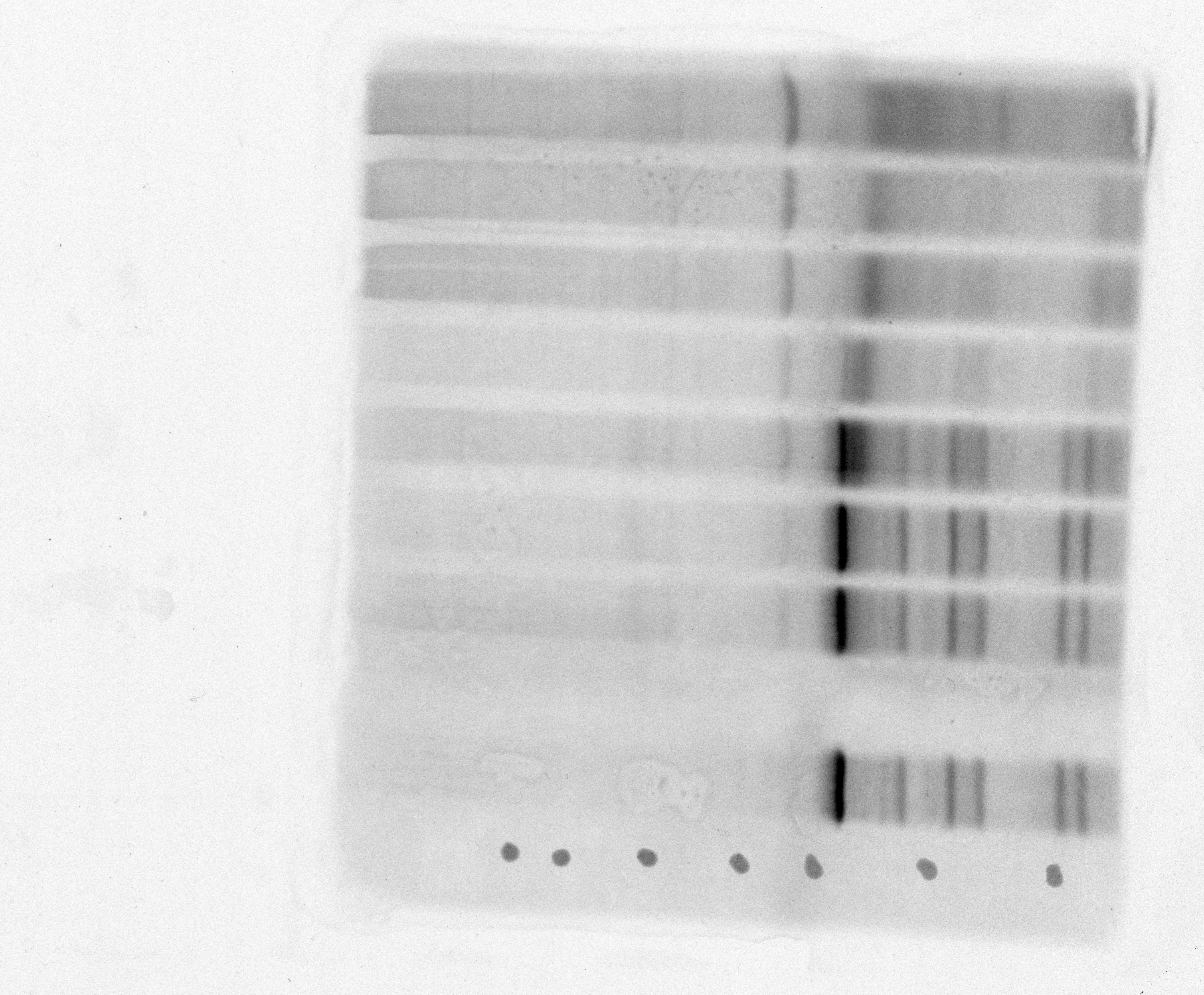

Supplement: Supplementary file 2 — Source Data for Expanded View [file EMBJ-36-693-s002.zip › EMBOJ-95336_EV_Source_Data/FigEV1/EV1A.tiff]

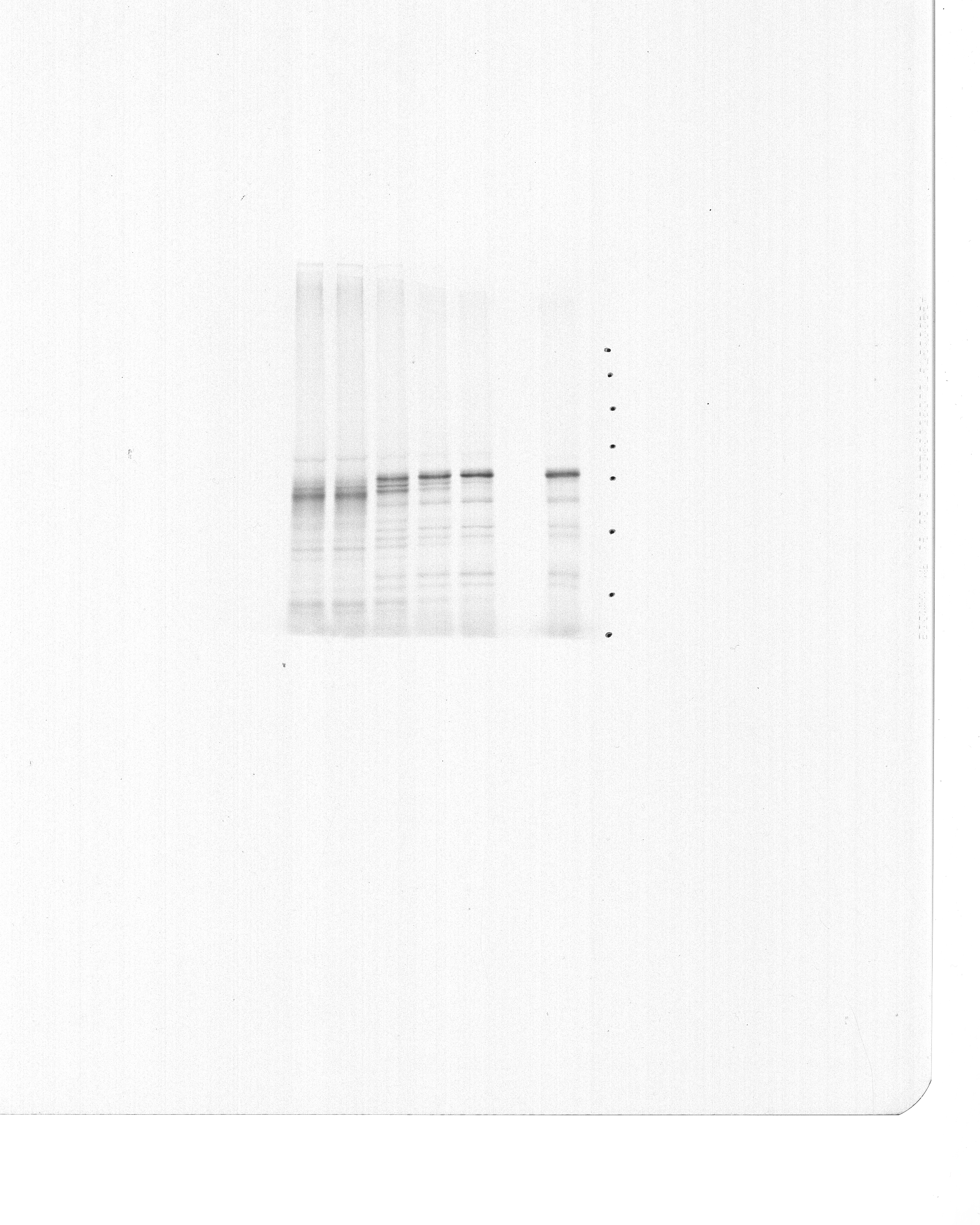

Supplement: Supplementary file 2 — Source Data for Expanded View [file EMBJ-36-693-s002.zip › EMBOJ-95336_EV_Source_Data/FigEV1/EV1B.tiff]

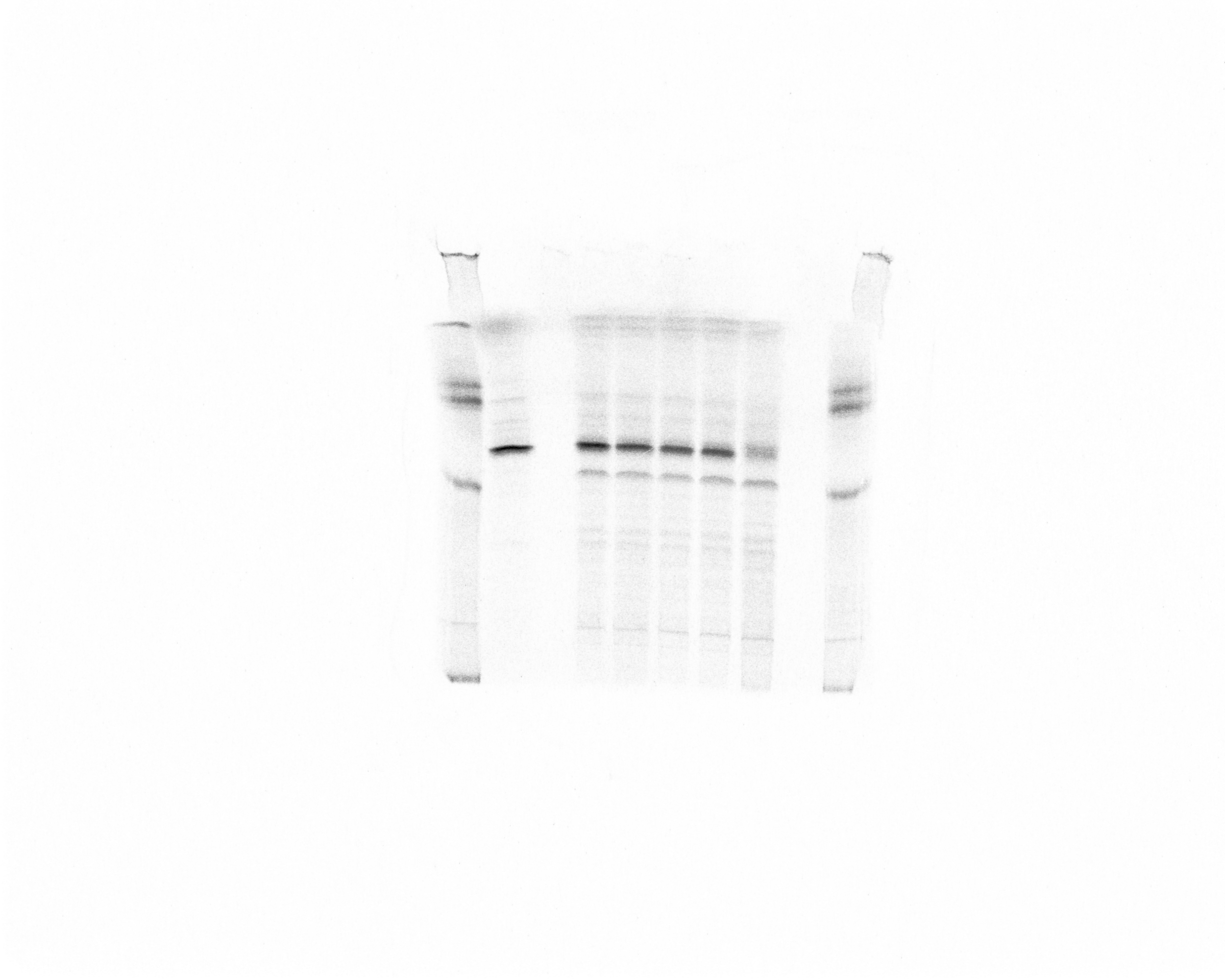

Supplement: Supplementary file 2 — Source Data for Expanded View [file EMBJ-36-693-s002.zip › EMBOJ-95336_EV_Source_Data/FigEV1/EV1C.tiff]
